# Low-cost 3D-printed mask system for versatile selective sample deposition on *in situ* TEM chips

**DOI:** 10.1016/j.ohx.2025.e00663

**Published:** 2025-06-04

**Authors:** Deema Balalta, Sara Bals

**Affiliations:** Electron Microscopy for Materials Science (EMAT), University of Antwerp, Groenenborgerlaan 171, 2020 Antwerp, Belgium

**Keywords:** Liquid cell transmission electron microscopy, MEMS chips, Shadow mask, Selective deposition, 3D-printing

## Abstract

*In situ* liquid electron microscopy has emerged as a powerful technique for studying dynamic processes at the nanoscale. However, selective deposition of samples on *in situ* biasing MEMS chips is far from straightforward due to the relatively small area of the electron-transparent window and the compact design of the three electrodes. This is particularly challenging for samples dispersed in solvents or those fabricated through physical vapor deposition. Here, we address these challenges by proposing a simple, low-cost, 3D-printed loading stage with an integrated mask system. Our design enables controlled deposition, as demonstrated by the successful deposition of AuPdPt nanoparticles from liquid suspension, sputtered Au clusters, and a cluster-based Au thin film onto the working electrode. The design can be easily fabricated in any electron microscopy lab, making it accessible and adaptable to various MEMS *in situ* chips and sample types.

## Graphical abstract

**Figure:**
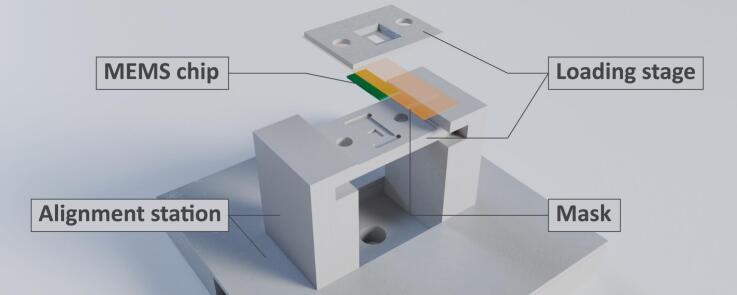

Specifications table

**Table:** 

Hardware name	Mask system for selective sample deposition on MEMS chips
Subject area	• Engineering and materials science
Hardware type	• Mechanical engineering and materials science
Closest commercial analog	• Shadow Masking setup from Protochips
Open source license	CC BY-SA 4.0 (Creative Commons Attribution-ShareAlike 4.0 International)
Cost of hardware	Approx. 10 EUR
Source file repository	https://doi.org/10.5281/zenodo.14826547

## 1 Hardware in context

**Fig. 1:**
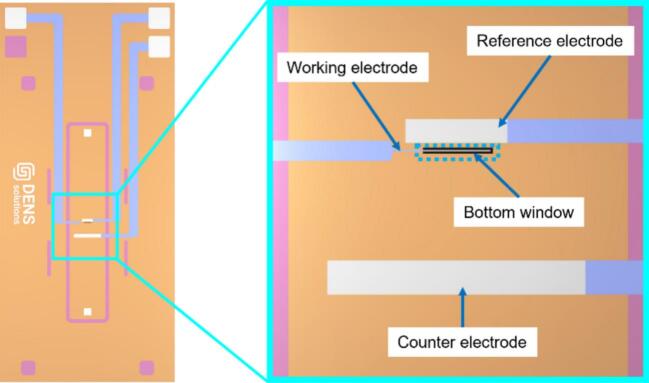
Schematic drawing of liquid-biasing bottom chip used in the DENSsolutions Stream holder system, including a magnified view of the three electrodes. The targeted deposition area, where the working electrode overlaps with the SiN_x_ window, is highlighted by the dotted blue rectangle. (For interpretation of the references to colour in this figure legend, the reader is referred to the web version of this article.)

*In situ* liquid cell transmission electron microscopy (LCTEM) enables the observation of dynamic processes at the nanoscale in liquid environments, offering valuable insights across fields such as materials science, electrochemistry and biology. In electrochemical studies, LCTEM is often enabled using micro-electromechanical system (MEMS) chips that feature electron-transparent SiN_x_ membranes. These chips provide a compact three-electrode system within the nanoreactor, but the small size and delicate nature of the setup present significant challenges for sample deposition prior to TEM measurements. Fig. 1 shows a schematic drawing of a liquid-biasing MEMS chip from DENSsolutions Stream holder system, illustrating the three electrodes layout and the target deposition area of working electrode (WE), which overlaps with the SiN_x_ window.

A reliable method for controlled sample deposition is essential for accurate *in situ* TEM studies, particularly for immobilizing catalysts on the WE without causing electrical shorts. For nanoparticle-based catalysts, conventional TEM sample deposition methods, such as drop-casting, are unsuitable due to the confined geometry of the biasing chip, where the distance between the WE and the reference electrode (RE) is less than 20 μm. Drop-casting involves applying a small volume of solution on the chip surface and allowing it to dry. However, this often leads to huge amounts of sample deposition across all the electrodes, potentially affecting their uniformity and functionality. Additionally, drop-casting can cause the formation of unwanted aggregates, which may interfere with the study or introduce electrical shorts. Without specialized nanocapillary systems [1], it is very challenging to precisely immobilize catalyst nanoparticles on the surface of the WE. For thin film-based catalysts, such as those prepared via physical vapor deposition (PVD) [2], existing techniques fall short. One common approach to study the cross-section of thin films is preparing TEM lamellae using focused ion beam (FIB) milling, which involves slicing a very thin section of the film to observe its structure in TEM. However, mounting it onto the WE of the biasing chip is not ideal. The fragility of the 50 nm-thick SiN_x_ window makes it vulnerable to damage from FIB’s high energy, and the thickness of the lamella edges often exceeds the nanoreactor's permissible dimensions, preventing a proper seal. A more suitable approach would be to deposit the thin film catalysts directly onto the WE of the chip. This would require high-precision deposition, which can be achieved using a shadow mask. While such systems exist to enable this kind of deposition [3], they are typically available only for certain brands of chips, limiting their general accessibility.

To overcome the limitations mentioned above, we present here a simple, low-cost 3D-printed loading stage and mask system designed to address these challenges. Our solution enables precise, high-resolution deposition of samples, such as AuPdPt nanoparticles, and sputtered cluster-based Au thin film onto the working electrode of MEMS biasing chips. This design is accessible, easy to fabricate, and adaptable to different *in situ* MEMS chips, providing a practical tool for advancing LCTEM studies by ensuring more reliable sample preparation.

## 2 Hardware description

**Fig. 2:**
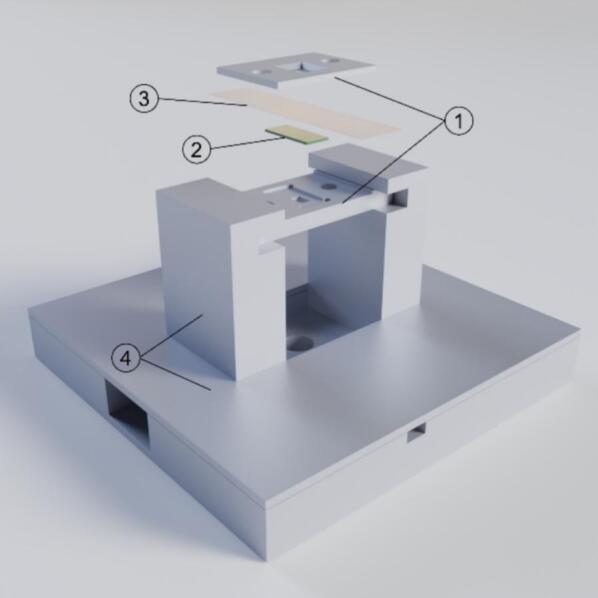
The basic structure of the 3D-printed loading stage mounted on alignment stage showcasing the MEMS chip and shadow mask placement. The numbered components are as follows: 1. loading stage 2. MEMS chip 3. shadow mask 4. alignment station.

The shadow mask system for selective deposition consists of three main components: (1) the loading stage, which houses the MEMS chip and the mask; (2) the shadow mask, featuring a precisely fabricated opening (200 × 20 μm); and (3) the alignment station, designed to facilitate accurate alignment of the shadow mask opening with the working electrode of the biasing chip. A 3D rendering of the system components is shown in Fig. 2.

**Fig. 3:**
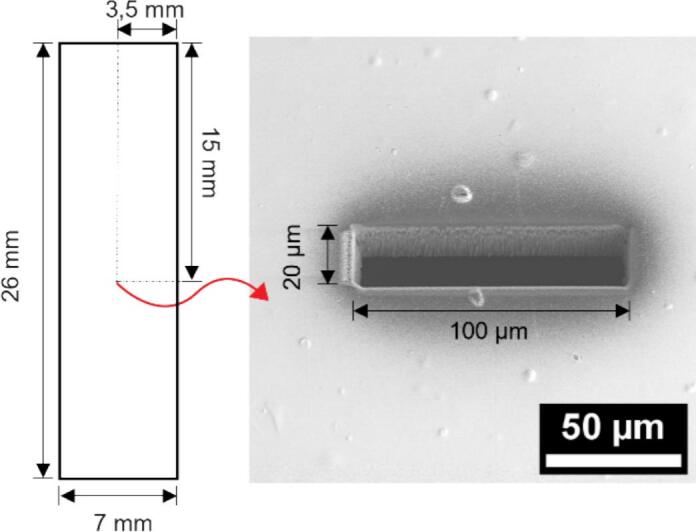
2D drawing of the mask design with the central opening, shown as SEM image indicated by the red arrow. (For interpretation of the references to colour in this figure legend, the reader is referred to the web version of this article.)

The loading stage is a two-part apparatus: the bottom part houses the MEMS chip securely within a precision-fit cavity, while the top part acts as a lid that holds both the chip and the shadow mask between the two parts. Threaded inserts in the bottom part allow screws to fasten the assembly, ensuring that all components remain stationary. This feature maintains the alignment of the mask during the sample deposition process, providing reliability and repeatability. The shadow mask is made of Kapton foil (26 × 7 mm, 25 μm thick) coated with a 120 nm layer of Au on both sides. It features a precisely fabricated opening that can be adjusted depending on the user’s needs, such as matching the dimensions of the electron-transparent window (200 × 10 μm) or the WE (200 × 3 μm). For effective deposition, the opening should be sufficiently wide to expose the target area, ensuring complete coverage and easier alignment. In our case, we used a 200 × 10 μm mask opening as the minimum size (approximately twice the width of the previously used 5 μm-wide working electrode). The opening is created using FIB milling, which ensures the precision required to achieve the desired dimensions and depth (25 μm), critical for effective selective deposition. Fig. 3 shows the simple design of the mask and a scanning electron microscope (SEM) image of the mask opening. The alignment station supports the loading stage under a light microscope, allowing high-magnification imaging for precise alignment of the shadow mask opening with the working electrode of the MEMS chip. Once aligned, the screws of the loading stage are tightened to secure the configuration. The loading stage and alignment station were fabricated using a 3D printer with sufficient spatial resolution to accommodate the MEMS chip dimensions (approximately 10.3 × 5 mm), making it a suitable and cost-effective choice for the application. Polylactic Acid (PLA) was selected for its affordability, accessibility, and compatibility with the desired design specifications. Our shadow mask provides the following features:

• Low-cost and accessible fabrication using 3D-printing and common materials.
• High precision selective deposition with a 200 × 10 μm mask opening.
• Versatile design adaptable to various MEMS chip configurations.
• Reliable alignment process facilitated by a dedicated alignment station.
• Open-source design, enabling customization for novel applications.

## 3 Design files summary

The design files include all documents required to fabricate and assemble the shadow mask system. These files cover 3D-printable components, reference images, an instructional video, and a bill of materials.

**Table:** 

**Design file name**	**File type**	**Open source license**	**Location of the file**
Loading_Stage_Base.stl	STL	CC BY-SA 4.0	https://doi.org/10.5281/zenodo.14826547
Loading_Stage_Top.stl	STL	CC BY-SA 4.0	https://doi.org/10.5281/zenodo.14826547
Alignment_Station_Base.stl	STL	CC BY-SA 4.0	https://doi.org/10.5281/zenodo.14826547
Alignment_Station_Lid.stl	STL	CC BY-SA 4.0	https://doi.org/10.5281/zenodo.14826547
Alignment_Station_Arm.stl	STL	CC BY-SA 4.0	https://doi.org/10.5281/zenodo.14826547
Printed_Parts_layout.png	Image	CC BY-SA 4.0	https://doi.org/10.5281/zenodo.14826547
Assembled_Parts.png	Image	CC BY-SA 4.0	https://doi.org/10.5281/zenodo.14826547
Mask-alignment.mp4	Video	CC BY-SA 4.0	https://doi.org/10.5281/zenodo.14826547
BOM.xlsx	Excel	CC BY-SA 4.0	https://doi.org/10.5281/zenodo.14826547

## 4 Bill of materials summary

The majority of components used in the building of this device were sourced from materials available in the lab at no additional cost. The primary cost was the purchase of the Kapton foil, but only a small piece (26 × 7 mm) was used for the mask. Therefore, the cost associated with this material is minimal and does not significantly impact the overall expense of the device. Additionally, while the 3D printing was done in our lab at no additional cost for the filament, we referenced the university's 3D printing service, which charges based on the cost of PLA filament. As for the gold sputtering, the process was carried out by the researcher herself, and the only associated cost was the gold. For all the masks tested, the total amount of gold used was less than 500 ng, resulting in a negligible cost. The complete bill of materials can be found on the Zenodo repository. The accompanying Excel document includes a detailed list of the components used and their approximate costs, where applicable.

## 5 Build instructions

Mask

• Cut Kapton foil strips to the dimensions of 26 × 7 mm.
• Cover both sides of the Kapton foil with conductive metallic layer, we used 120 nm layer of gold using a sputter coater. This coating is essential for making the foil conductive, which prevents charge accumulation during FIB milling and ensures precise, stable cutting of the mask features.
• Using FIB milling, carefully cut the mask opening to the desired dimensions (e.g., as small as 200 × 10 μm, or other desired sizes). The milling parameters used were 9.3nA current at 30 kV acceleration voltage. Ensure that the milling is done perpendicular to the mask’s length, which is crucial depending on the design of the MEMS chip’s working electrode. You can check the mask design in Fig. 3.

Loading Stage and Alignment Station

• 3D print the loading stage and alignment station parts using the STL files available in the design file summary (ensure that the Alignment_Station_Arm.stl is printed twice). The printer used was an Ultimaker 3 Extended with 0.1 mm resolution, 60 % infill intensity, and 100 % print speed.
• After printing, check the fit of the chip in the cavity on the bottom part of the loading stage. Ensure that the chip sits flush with the surface.
• If necessary, lightly sand the cavity edges with 220 grit sandpaper to improve the fit. The cavity dimensions can be adjusted before printing to fit the user’s chip size, allowing for customization based on specific chip requirements.
• Additionally, the height of the alignment station arms can be adjusted based on the focal plane of the light microscope in use, ensuring proper alignment during the process.
• For assembling the loading stage, insert the threaded inserts for the screws into the designated holes in the bottom part of the loading stage. Use a heated iron tip set to 250 ˚C to heat the metal insert. The heat softens the PLA, allowing the insert to be smoothly pressed into the hole. Once in place, the insert cools and securely bonds to the PLA, creating a stable connection for the screws.
• Connect a basic LED light circuit to fit inside the alignment stage base, then secure the base cover to seal it in place. While it is possible to glue the cover and the arms of the station (which hold the loading stage), this approach was avoided to allow for easier handling of the loading stage when placing it in and removing it from the light microscope base during the mask alignment process. The design files summary shows 3D images of the individually printed parts and assembled loading stage and alignment station.

## 6 Operation instructions

**Fig. 4:**
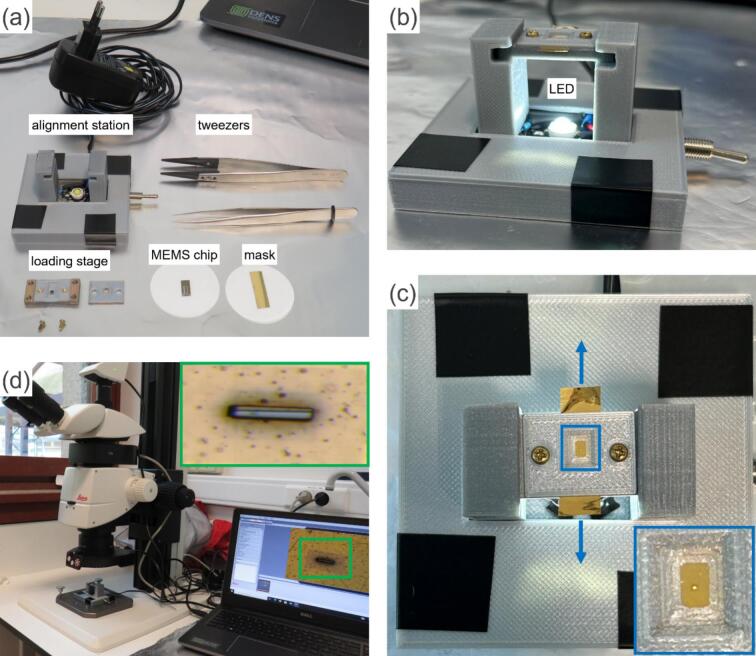
Assembly and alignment process for biasing chip and mask, (a) parts layout prior to assembly, (b) loading stage mounted onto the alignment station, (c) initial mask alignment, with an inset showing a magnified area where light passes through the mask opening, and (d) fine-tuning alignment using M125 C Leica microscope, with an inset showing the working electrode (the black line) through the opening.
• Mount the loading stage onto the alignment station arms as shown in Fig. 4b and turn on the LED light. The mask’s opening is not visible to the naked eye. For the initial alignment, gently move the mask back and forth using plastic-tipped tweezers, as indicated by the blue arrows in Fig. 4c. Make small adjustments until you see light passing through the mask. This indicates that the SiN_x_ window and the mask opening are initially aligned, allowing the light to pass through.
• Now, move to the light microscope (Fig. 4d) for the fine tuning of the alignment to ensure the mask is fully aligned with the working electrode of the biasing chip. Refer to the mask alignment video from the design file summary for further guidance on the process.
• Once the alignment is completed, tighten the screws of the loading station, to hold the mask securely in place and allow for safe, precise selective deposition of the sample.

• Fig. 4a shows laid out parts required for the assembly. Begin by loading the biasing chip and the mask inside the loading stage, secure the screws and tighten them leaving about a quarter of a turn from full tightening. This slight looseness allows for small adjustment in the mask’s position to align it with the working electrode.

## 7 Validation and characterization

**Fig. 5:**
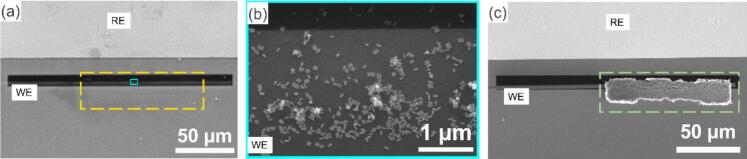
SEM images of (a) *in situ* biasing chip, showing the SiN_x_ window containing the working electrode and the reference electrode, with the deposited AuPdPt sample contained within 102 × 24 μm borders (highlighted by the yellow dashed rectangle), (b) a magnified view of the area in sky blue rectangle, showing the nanoparticles deposited on the working electrode, and (c) *in situ* biasing chip with a drop-casted sample of carbon black with Pt nanoparticles, contained within 100 × 22 μm borders (highlighted by the green dashed rectangle). (For interpretation of the references to colour in this figure legend, the reader is referred to the web version of this article.)

First, we evaluated the mechanical safety of the chip during the mounting and mask alignment process. The material selection of the mask was critical to ensure its durability, address the fragility of the SiN_x_ window (200 × 10 μm, 50  nm thick), and enable the precise definition of the mask opening feature. Kapton foil with a thickness of 25 μm was chosen because of its excellent chemical resistance, and mechanical properties, making it suitable for the fine-scale fabrication required for this application. Its small thickness allows for a tight fit within the two pieces of the loading stage without adding significant bulk, and enables the mask to cover the required area of the chip accurately. Additionally, the flexibility of the Kapton foil helps in protecting the fragile SiN_x_ window from breaking during the assembly by ensuring a uniform compression when the loading stage screws are tightened. This distributes the pressure evenly across the chip, reducing the risk of damage to the delicate membrane. We have tested the performance of the shadow mask system for two sample deposition techniques:

(**1) Liquid drop-casting,** three 2 µl droplets of AuPdPt nanoparticles suspended in water were deposited at the surface of the mask (opening size: 100 × 20 μm) and left to dry under the fume hood between each droplet. Upon examining the biasing chip via SEM, we could locate the deposition area of the nanoparticles on the chip (Fig. 5a), which was contained within (102 × 24 μm). This is a few microns larger than the mask opening dimensions, indicating a small bleeding effect. An extremely low concentration of nanoparticles was observed in the masked area, but their presence was minimal and didn’t extend to the reference electrode, ensuring its protection. The sample used for this test was highly concentrated to exaggerate the bleeding effect and evaluate the mask's effectiveness. For future *in situ* biasing experiments, however, samples will be diluted, and fewer drop-casting steps will likely minimize the spread of the sample beyond the mask opening. Furthermore, we extended our investigation by testing a sample suspended in ethanol (carbon black with Pt nanoparticles), which showed similar results to those observed with water, with a deposition area of (100 × 22.5 μm) (Fig. 5c), indicating consistent behavior across different liquids. While the system was successfully validated for wet casting, it is expected to work similarly for dry casting, though this has not yet been tested. Fig. 5 shows the successful wet casting deposition on the working electrodes of the biasing chips.

**Fig. 6:**
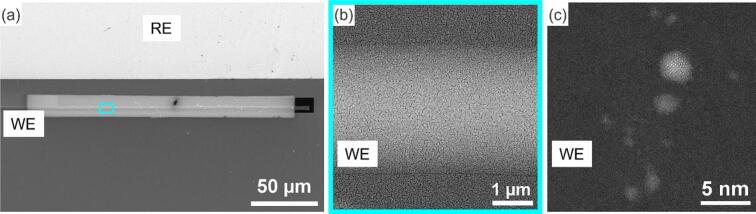
(a) SEM image of an *in situ* biasing chip, showing the sputtered Au thin film contained within the mask borders (200 × 15 μm) after 200 s of sputtering. High resolution HAADF STEM images of (a) the area in sky blue rectangle in (a), showing Au thin film covering the glassy carbon WE and the surrounding SiN_x_ window (b) a second chip, showing Au clusters deposited on the WE after 3 s of sputtering. (For interpretation of the references to colour in this figure legend, the reader is referred to the web version of this article.)

**(2) For physical vapor deposition (sputtering)**, we used ACE600 Leica magnetron sputter coater with a gold target and applied 30 mA for 3 and 200 s. The loading stage was positioned facing the gold target during the deposition process. Upon examining the WEs of the biasing chips, we observed a low density of Au clusters after the 3-second deposition, whereas a continuous Au thin film after 200 s. Fig. 6a shows SEM image of the Au thin film deposited using (200 × 15 μm) mask opening, Fig. 6b&c include high resolution high angle annular dark field scanning transmission electron microscope (HAADF STEM) images of the chips corresponding to the 200-second and 3-second depositions, respectively.

## 8 Mask optimization and outlook

In the process of optimizing the mask design, several mask sizes and configurations were tested. The smallest mask opening width tested was 10 μm, but aligning such a fine feature was difficult due to the manual alignment process. Although precise alignment was possible, it was time-consuming and required significant effort. In contrast, masks with 20 µm width (approximately twice the width of the chip window) were much easier to align. During our tests, most of these wider openings were almost perfectly aligned during the light-guided alignment step.

**Fig. 7:**
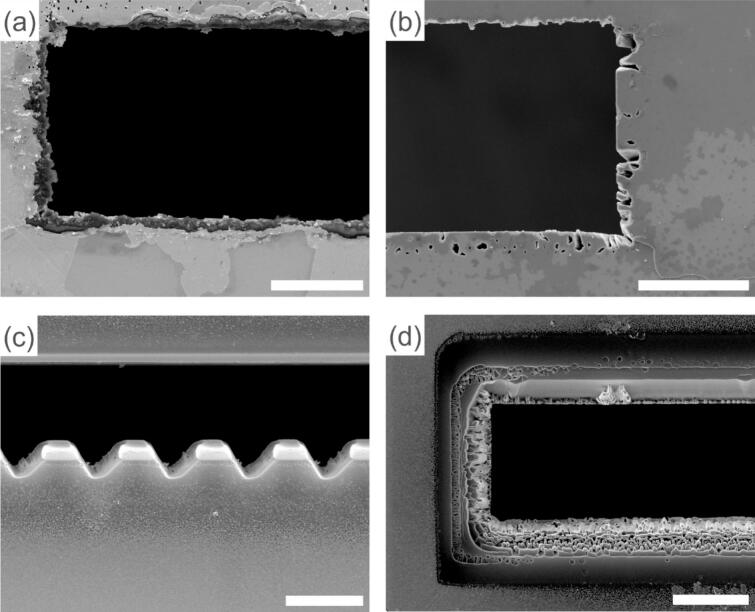
SEM images of investigated mask edge morphologies: (a) rough edge from low milling current, (b) smooth edge from high milling current, (c) zigzag edge to disrupt surface tension, and (d) ramped edge to reduce shadowing effects. Scale bars: 10 μm.

One of the main challenges in this selective deposition is overcoming surface tension effects, which can prevent the sample solution from flowing through the mask opening and fully contacting the MEMS chip. This is especially problematic when using water as a solvent, as its high surface tension can cause the liquid to remain on the mask surface, rather than flowing through the opening to the electrode. To address this, further experiments were conducted to optimize the edge configuration of the opening. When the opening borders were milled at low current (9.3 nA), a rough edge was formed (Fig. 7a), which disrupted the surface tension of the drop-casted solution, allowing the nanoparticles to flow through the mask and onto the chip, as demonstrated in Fig. 5. In contrast, milling at higher current (21 nA) produced smooth, sharp edges (Fig. 7b), that hindered the deposition. Despite using a larger mask opening (200 × 20 μm), no nanoparticles were deposited on the chip, indicating that smooth edges were insufficient in breaking the surface tension. A zigzag edge design on one side of the mask was also tested (Fig. 7c) to aid in breaking the surface tension. However, it proved less effective, with only a few nanoparticles passing through the opening. While the zigzag structure was intended to disrupt the liquid interface, its morphology might not have been pronounced enough to overcome surface tension effects in the same way that edge roughness did. Additionally, a ramped edge design was tested (Fig. 7d), which helped mitigate the shadowing effect during sputtering. This modification allowed for a larger collection angle of sputtered clusters, improving the uniformity of deposition.

These results highlight that the ability of the liquid to flow through the mask opening is primarily governed by the roughness of the mask opening edges, rather than the dimensions of the opening itself. While increasing the mask size might intuitively seem to facilitate deposition, our findings show that surface tension effects prevent effective liquid transport unless the mask edges have sufficient roughness to disrupt the liquid interface. This emphasizes the importance of controlling edge morphology, over simply enlarging the mask opening size to overcome capillary effects. Fig. 7 presents SEM images of the various mask configurations investigated, illustrating the effects of different milling techniques on the mask structure.

Looking ahead, we envision the potential for batch production of these masks. Currently, the masks are single-use due to challenges with cleaning, a femtosecond laser cutter could be used to achieve the high-resolution mask openings without inducing heat-related damage to the Kapton foil. Femtosecond laser pulses are so short that they do not allow sufficient time for heat transfer effects to occur. This would eliminate the need for the gold coating, simplifying both fabrication and alignment due to Kapton’s transparent nature, further reducing the complexity involved in mask setup. This system is highly adaptable and can be used for any *in situ* chip design where selective deposition is required. By adjusting the bottom part of the loading stage, it can be modified to accommodate chips of various sizes, making the system versatile for different applications.

## CRediT authorship contribution statement

**Deema Balalta:** Writing – review & editing, Writing – original draft, Visualization, Validation, Methodology, Investigation, Formal analysis, Data curation, Conceptualization. **Sara Bals:** Writing – review & editing, Supervision, Project administration, Funding acquisition.

## Declaration of competing interest

The authors declare that they have no known competing financial interests or personal relationships that could have appeared to influence the work reported in this paper.

The authors further declare that this design is protected, but it is freely available for academic and research use under the CC BY-SA 4.0 license.
